# Guided Removal of Long and Short Fiber Posts Using Endodontic Static Guides: A Case Report

**DOI:** 10.1002/ccr3.70438

**Published:** 2025-04-18

**Authors:** Sahar Shafagh, Mamak Adel, Atiyeh Sabzpai

**Affiliations:** ^1^ Department of Endodontics, School of Dentistry Qasvin University of Medical Science Qasvin Iran; ^2^ Department of Endodontics, Dental Caries Prevention Research Center Qasvin University of Medical Science Qasvin Iran; ^3^ Department of Prosthodontics, School of Dentistry Qasvin University of Medical Science Qasvin Iran

**Keywords:** cone‐beam computed tomography, fiber posts, guided endodontics, post removal

## Abstract

Fiber posts are commonly used in restorative dentistry. However, their removal poses significant challenges, including the risk of root fractures, deviations, or perforations. Recently, guided endodontic techniques have emerged as a conservative and precise approach for fiber post removal, though clinical applications remain underexplored.

## 1 Introduction

In recent decades, fiber posts combined with composite cores have gained widespread popularity for the restoration of teeth [1]. The primary advantages include superior flexibility and aesthetics compared to metal posts, along with a modulus of elasticity (stiffness) that closely matches dentin. Additionally, failure modes typically involve debonding of the composite core, which is considered advantageous since it can be repaired [2, 3]. Fiber posts are bonded within the root canal using adhesive materials such as composite resin or glass ionomer cements, which are notoriously challenging to remove [2]. However, removal may be necessary due to endodontic treatment failure or prosthetic issues. Reports suggest that fiber posts can be fragmented and extracted using a microscope in combination with ultrasonic tips, long‐shank round burs, and/or specialized removal tools. The procedures are technically demanding, requiring meticulous care to avoid root fractures, significant deviations from the root axis, crack propagation, or root perforations. Moreover, they are time‐consuming and highly dependent on the clinician's skill [4, 5, 6, 7]. Given these challenges, there is a need for improved techniques and innovative solutions to address this clinical problem effectively [8], such as the use of robotic technology where adequate inter‐arch space is available or the application of the guided technique [9].

Currently, guided endodontics is widely used in managing calcified root canals [10], for the removal of root canal barriers such as mineral trioxide aggregate [11], retrieval of a separated intracanal file [12], and fiberglass posts [13]. A systematic review of in vitro studies demonstrated that the guided technique for fiber post removal and accessing through MTA preserves more tooth structure. However, when used for the removal of separated files, it may lead to a higher risk of iatrogenic errors compared to the freehand technique [14]. This technique employs two types of endodontic guides: dynamic and static. Dynamic guides are real‐time intraoperative navigation devices that use cameras to ensure precise excision execution of a preplanned treatment strategy [15]. Static guides are three‐dimensional resin templates. Their fabrication requires a cone beam computed tomography (CBCT) scan and an optical impression of the treatment area [16, 17].

Endodontic static guides have gained popularity as a conservative and precise method, particularly for managing calcified canals. While their use for fiber post removal was introduced recently, their clinical application remains relatively underexplored. The technique offers a significant advantage by preventing tooth extraction during fiber post removal, but most existing studies are laboratory‐based, with limited clinical data available to assess its success [18]. Therefore, the present case report utilizes the static‐guided endodontic technique to remove two fiber posts from the compromised maxillary central and canine incisors of a middle‐aged patient.

## 2 Case History/Examination

**FIGURE 1:**
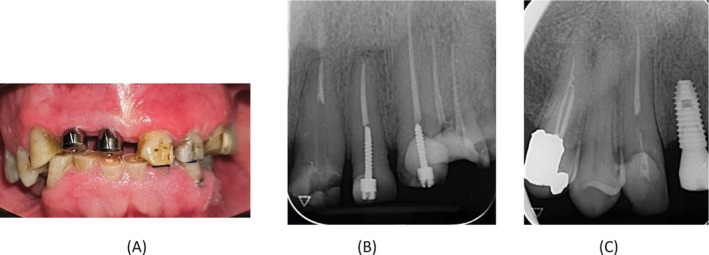
(A) Preoperative clinical photograph. (B) Perioperative periapical radiograph of tooth #9 showing a long fiber post extending into the apical third of the canal. (C) Perioperative periapical radiograph of tooth #6 showing a short root canal filling.

A 54‐year‐old male patient with no medical history presented to the postgraduate endodontic clinic at Qazvin University of Medical Sciences, referred by the prosthetics department for the removal of fiber posts in teeth #6 and #9. This was necessitated as part of a comprehensive full‐mouth reconstruction plan to address dental attrition. The patient reported a history of pain in tooth #6, which was tender to percussion, whereas tooth #9 showed no such tenderness. Radiographic evaluations showed a short filling in tooth #6 and a long fiber post extending into the apical third of the canal in tooth #9 (Figure 1).

## 3 Methods

**FIGURE 2:**
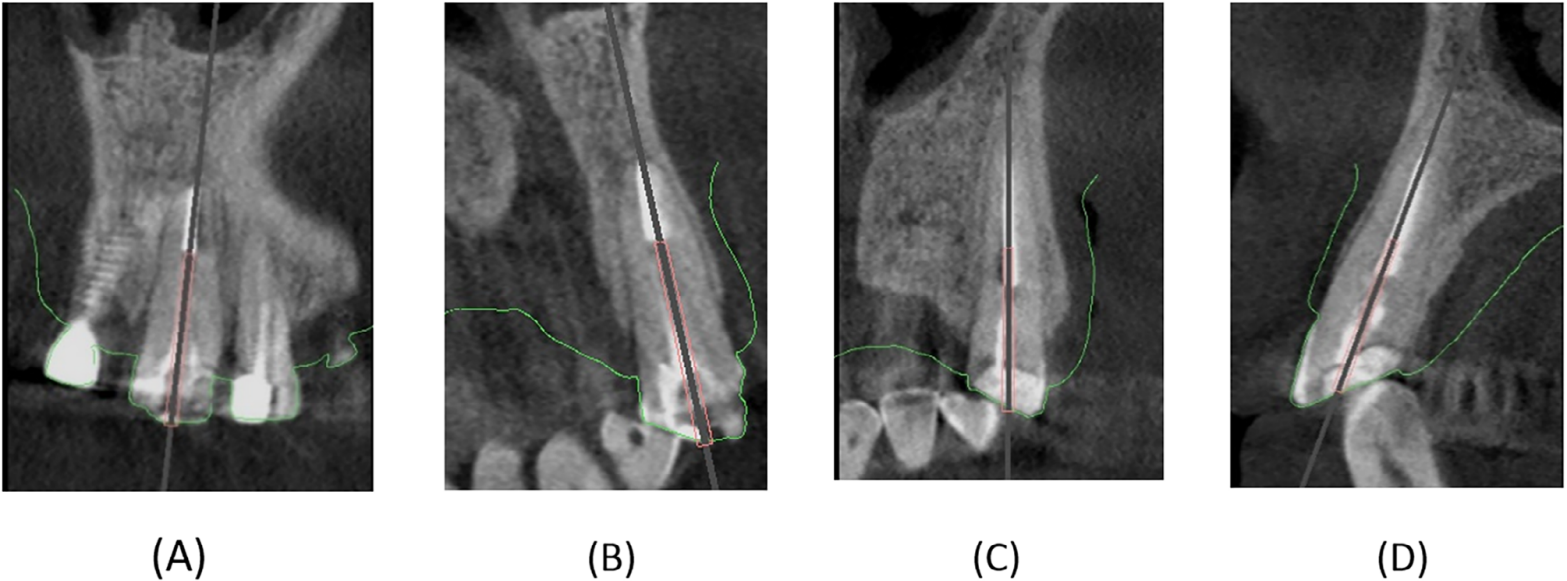
(A) Virtual planning for fiber post removal in the coronal plane of tooth #9. (B) Virtual planning for fiber post removal in the sagittal plane of tooth #9. (C) Virtual planning for fiber post removal in the coronal plane of tooth #6. (D) Virtual planning for fiber post removal in the sagittal plane of tooth #6.

Given the critical importance of preserving teeth #6 and #9 for prosthetic treatment, and the inherent risks associated with fiber post removal, a 3D‐printed guide was proposed to the patient, and informed consent was obtained. A CBCT scan was performed for teeth #6 and #9, and the surrounding structures (Planmeca Promax 3D system, Helsinki, Finland) operating at 84 kV, 9 mA, with a field of view of 8 × 8 cm, and a voxel size of 0.15 mm. Also, an intraoral scan was acquired using the Medit i500 (South Korea). The obtained image data, in digital imaging and communications in medicine (DICOM) and standard tessellation language (STL) formats, were imported into planning software (Nemotec, Spain). During the planning phase, a path was designed to accommodate a size 1 Munce bur (CJM Engineering, Santa Barbara, CA, USA), considering the position of the fiber post (Figure 2). The virtual guides were subsequently printed using a 3D printer (HeyGears, Guangzhou, China) with a layer thickness of 50 μm. The guides were fabricated with a 3‐mm thickness and included guiding sleeves with an internal diameter of 1.3 mm and an external diameter of 5 mm for both cases.

**FIGURE 3:**
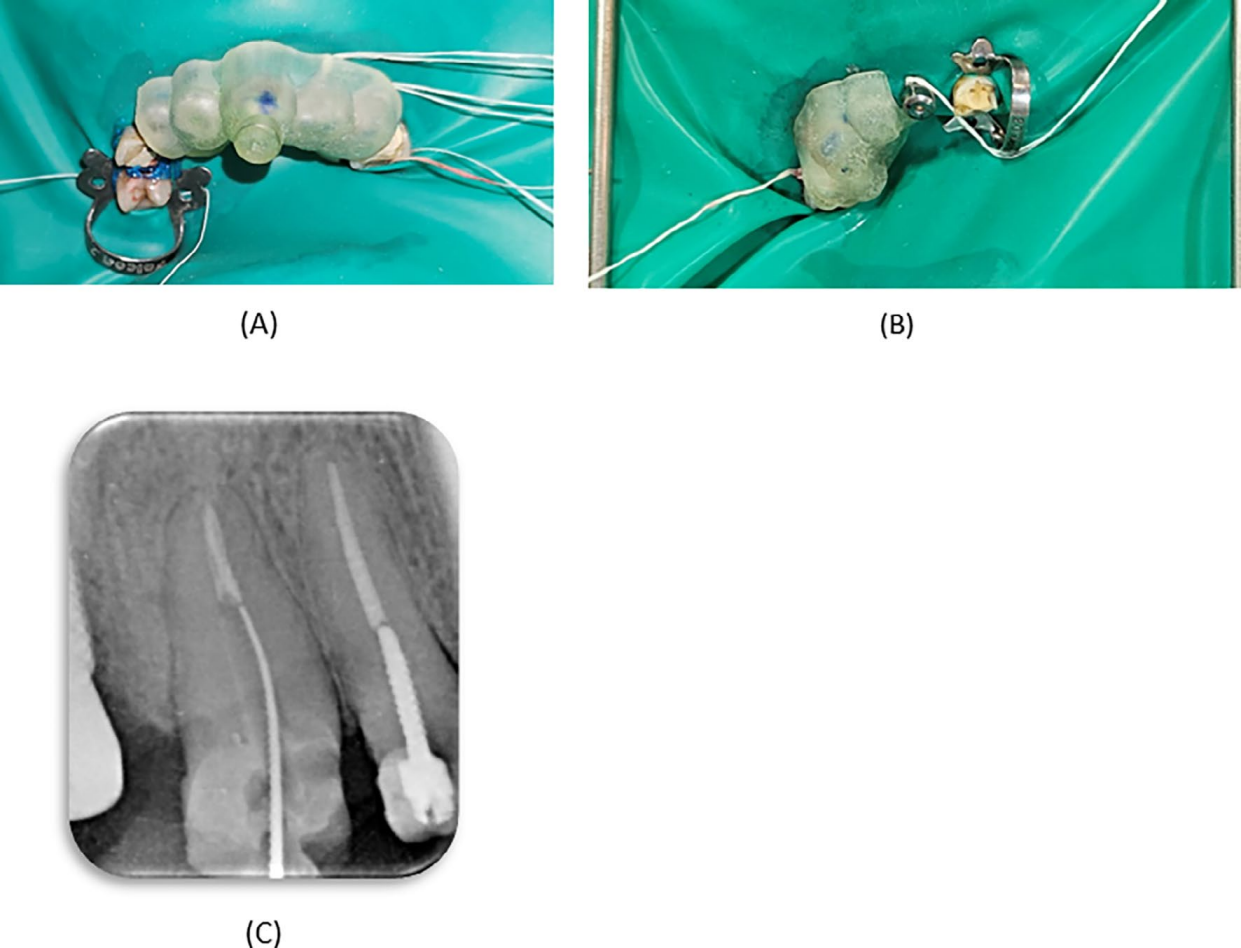
(A) The guide positioned on tooth #9. (B) The guide positioned on tooth #6. (C) K‐file reached the gutta‐percha in tooth #9.

On the second visit, a wash impression (Speedex Light Body, Coltene, USA) was taken to determine the precise fit of the guides. After administering local anesthesia, a rubber dam was placed, and the stability of the guide for tooth #9 was rechecked. Drilling was then performed under a microscope (MediWork, China) using a Munce bur in a controlled back‐and‐forth motion, with continuous irrigation to prevent overheating of the guide. After each 2 mm penetration, the guide was removed, the path was checked with a k file #15 (Mani, Japan) under magnification, and the canal was irrigated with normal saline. Given the gap between the fiber post and the canal wall, a K‐file reached the gutta‐percha before the bur completed the entire path to the gutta‐percha, which was confirmed with a radiograph (Figure 3). The remaining fiber post, considering the path of the K file, was removed with ultrasonic tips (E4s and E1s, Eighteeth, China) and U‐file (Denco, China). In the radiograph, some remnants of the fiber post seemed to be present on the mesial wall, but nothing was visible under the microscope. However, no further attempts were made due to the risk of removing additional dentin and weakening the root structure. For tooth #6, the same protocol was followed, but complete removal of the fiber post was achieved using the Munce bur. After the completion of drilling, the coronal part of gutta‐percha was visible under magnification. Both teeth were temporarily restored with Cavit (Cavisol, Golchai, Iran), and the patient was scheduled for the next appointment.

**FIGURE 4:**
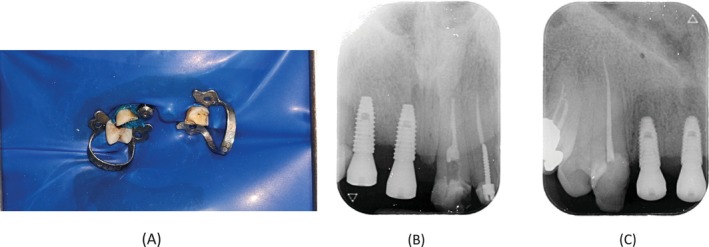
(A) Rubber dam placement during the retreatment session. (B) Final radiograph showing tooth #9 with temporary filling. (C) Final radiograph showing tooth #6 with temporary filling.

On the third visit, endodontic retreatment was performed for teeth #6 and #9. After administering local anesthesia, a rubber dam was placed. The restorative materials on the lingual surfaces of both teeth were removed to provide straight‐line access. Retreatment was performed using manual and retreatment rotary files (SP1, Fanta, China), accompanied by chloroform. Working lengths were confirmed using an apex locator (Woodpex V, Woodpecker, China) and verified radiographically. Canal preparation was then completed with rotary files (SP1, Fanta, China), accompanied by irrigation with sodium hypochlorite (NaOCl) and normal saline. The final irrigation protocol was performed using a sonic device (Easyinsmile, China), starting with 5.25% NaOCl and then followed by 17% EDTA and finishing with normal saline. The canals were obturated using the lateral condensation technique with an AH‐26 root canal sealer (Dentsply Sirona, Ballaigues, Switzerland). Finally, the teeth were temporarily restored with light‐cure glass ionomer cement (GC Fuji II LC, Tokyo, Japan) and referred for permanent restoration (Figure 4).

## 4 Conclusion and Results

The patient was referred to a prosthodontist for further treatment. During the follow‐up telephone call, no complications were reported.

## 5 Discussion

This case report highlights the successful use of static‐guided endodontic techniques to remove two fiber posts from teeth #6 and #9 in a middle‐aged patient requiring full‐mouth reconstruction. The procedure was performed to preserve the teeth for prosthetic rehabilitation while minimizing risks. The guided approach demonstrated high precision in fiber post removal, with minimal residual material and no significant damage to the surrounding tooth structure. The case also underscored the value of combining 3D printing technology, CBCT imaging, and intraoral scanning to achieve accurate planning and execution in complex endodontic procedures. In cases such as this, where fiber post removal is essential for crown reconstruction and intraradicular retention, challenges like perforation, dentin loss, and subsequent weakening of the tooth structure may arise [19]. To address these concerns, a guided technique was employed in this case, as it has been shown to reduce errors during clinical treatment [8, 20, 21].

Laboratory studies further support the efficacy of guided techniques. In this regard, Ito et al. conducted an ex vivo study to evaluate deviations in coronal, sagittal, and horizontal planes, as well as angular deviation during fiber post removal using guided versus freehand methods. They concluded that guides significantly reduced sagittal and horizontal deviations [22]. Similarly, Krug et al. compared conventional and guided techniques for the removal of fiber posts located in the apical third of root canals. They found that while both methods resulted in dentin loss and residual resin, the guided technique reduced dentin loss compared to the conventional approach [23]. Additionally, Yumin Wu et al. demonstrated that in molar teeth, the guided techniques resulted in less angular deviation and volume loss compared to the microscope and ultrasonic methods in fiber post removal [24], which is consistent with the finding of Wesley Fernandes Gonçalves et al. in their study comparing the guided technique and the conventional method for fiber post removal in premolar teeth [25].

In this case, despite ensuring the precise fit of the guide for tooth #9 by taking a wash impression, some resin residue remained [26]. This outcome highlights the common issue of a potential mismatch between the fiber post diameter and the bur used for removal. Residual cement and post fragments can be removed using ultrasonic tips under magnification, as was done in this case [23]. As Krug et al. suggest, in challenging cases, leaving some remnants of the post and resin is recommended to minimize damage to the tooth [23]. This approach was applied to tooth #9, where some resin and fiber post material remained on the mesial wall [23].

Despite its advantages, guided techniques have limitations. For instance, this method requires CBCT imaging, which involves a higher radiation dose compared to periapical radiographs. Nevertheless, as stated by the American Association of Endodontists: “CBCT should be used only when the patient's history and a clinical examination demonstrate that the benefits to the patient outweigh the potential risks.” This case can be considered an indication of the use of CBCT [27]. Additionally, there is a possibility of errors in intraoral scanning, which can be corrected by performing a wash impression to achieve precise seating. As with any guided procedure, the effectiveness depends on the clinician's ability to interpret imaging and execute the plan accurately. Finally, there is limited clinical data on the long‐term outcomes of guided fiber post removal, necessitating further research to validate its success in case series with long follow‐up.

In cases involving the removal of two fiber posts from two teeth, guides can be designed as either a single or double guide. According to a study by Dianat et al., there is no significant difference between these two methods. However, numerically, the double guide performed better in the coronal region, while the single guide showed better performance in the apical region [28]. For this case, we used a double guide because it was simpler for the laboratory to fabricate and reduced the likelihood of error during printing. In conclusion, the use of a 3D guide facilitates fiber post removal with fewer errors during treatment. The use of a guide is recommended in such cases, as it offers significant advantages in precision and tooth preservation. While certain limitations remain, such as the remaining resin and post in tooth #9 that we encountered in this case, future advancements in digital dentistry are likely to address these challenges and further enhance the effectiveness of guided techniques.

## Author Contributions

**Sahar Shafagh:** conceptualization, data curation, investigation, methodology, resources, software, validation, visualization, writing – original draft, writing – review and editing. **Mamak Adel:** conceptualization, data curation, investigation, project administration, resources, writing – original draft, writing – review and editing. **Atiyeh Sabzpai:** conceptualization, investigation, methodology, project administration, resources, supervision, validation, writing – original draft, writing – review and editing.

## Consent

Written informed consent was obtained from the patient to publish this report in accordance with the journal's patient consent policy.

## Conflicts of Interest

The authors declare no conflicts of interest.

## Data Availability

The data that support the findings of this study are available on request from the corresponding author. The data are not publicly available due to privacy or ethical restrictions.
